# A generalization of the Marmarou model

**DOI:** 10.1186/2045-8118-12-S1-P44

**Published:** 2015-09-18

**Authors:** Kaylan Raman

**Affiliations:** 1 Northwestern University, USA

## Introduction

The Marmarou model has fundamentally influenced the development of mathematical pressure-volume models of cerebrospinal fluid (CSF) dynamics. It is a non-linear ordinary differential equation which is solvable in closed-form. The solution provides valuable analytical insights into the nature of the circulatory dynamics of CSF. It would be useful to generalize the Marmarou model to a broader class of polynomials to expand its scope to other CSF and Hydrocephalus phenomena.

## Methods

The Marmarou model relating the temporal evolution of ICP in pressure-volume studies to infusions incorporates observed fluctuations in the ICP through a nonlinear ordinary differential equation (ODE) of second order whose structure is based on physical analogies between CSF dynamics and an electrical circuit. This ODE is extended from its quadratic structure to a general polynomial of order ‘n’.

## Results

An algorithm to solve the general polynomial ODE is developed. The solution to the original Marmarou model emerges from this algorithm as a special case. The solution for the general polynomial ODE is obtained from the algorithm which is shown to be easily implementable.

## Conclusions

The general polynomial Marmarou model expands the domain of applicability of the original one, thereby providing insights into a broader class of CSF phenomena. From a clinical perspective, these insights have implications for risk management of the patient for a large class of CSF and Hydrocephalus phenomena.

